# Supplementary material for: Membrane contact site detection (MCS-DETECT) reveals dual control of rough mitochondria–ER contacts
Source: J Cell Biol. 2023 Nov 10;223(1):e202206109. doi: 10.1083/jcb.202206109 (PMC10638097; doi:10.1083/jcb.202206109)
Supplement: SourceData FS3 — is the source file for Fig. S3. [file JCB_202206109_SourceDataFS3.pdf]

Source data for Supp Fig 3A

anti-Flag (Gp78)

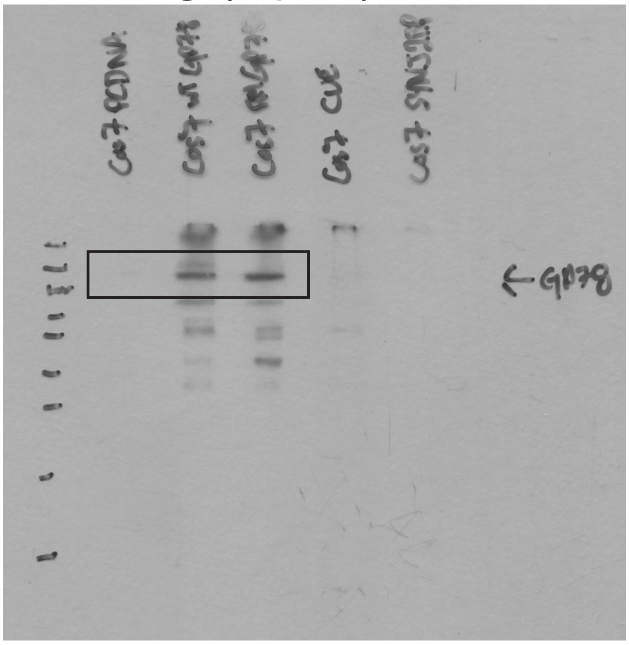

anti-β-actin

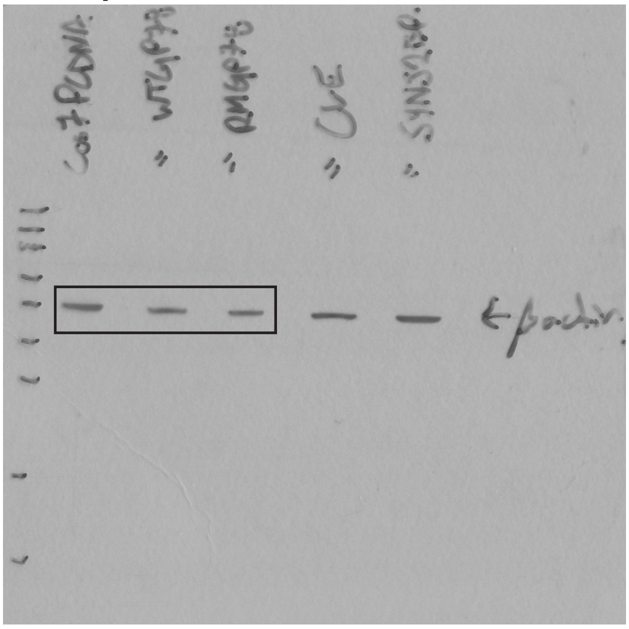

Cell lysates of COS-7 cells transfected with pcDNA3, Flag-Gp78 WT, Flag-Gp78 RM. SDS-PAGE were loaded from the left with lystaes of these samples in that order and Western blotted with anti-Flag (left) to detect Flag-Gp78 and anti-β-actin (right).

- Boxes show regions included in Supp Fig 3A.
- Molecular weight markers from top: 130, 100, 70, 55, 43, 34, 26, 17, 10 kDa are shown to the right.
- Gp78 migrates at ~78 kDa and β-actin at ~43 kDa.
