# Supplementary material for: Membrane contact site detection (MCS-DETECT) reveals dual control of rough mitochondria–ER contacts
Source: J Cell Biol. 2023 Nov 10;223(1):e202206109. doi: 10.1083/jcb.202206109 (PMC10638097; doi:10.1083/jcb.202206109)
Supplement: SourceData FS4 — is the source file for Fig. S4. [file JCB_202206109_SourceDataFS4.pdf]

Source Data for Supp Fig 4B

anti-RRBP1

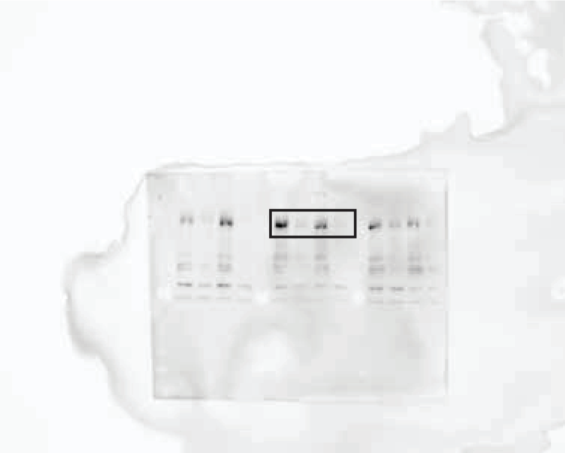

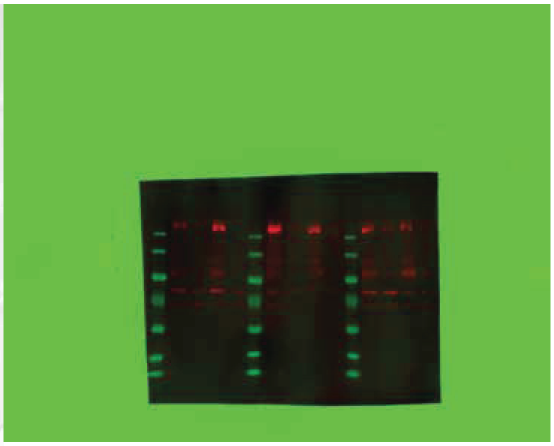

anti-β-actin

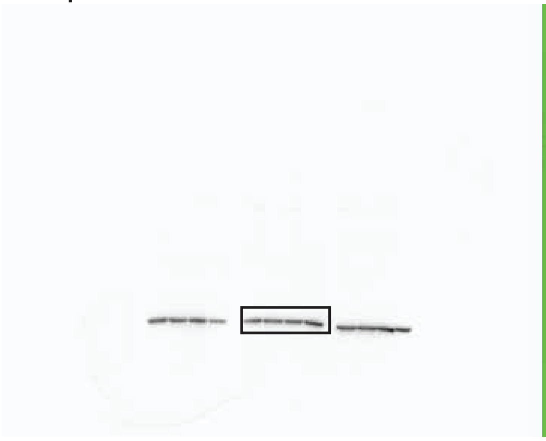

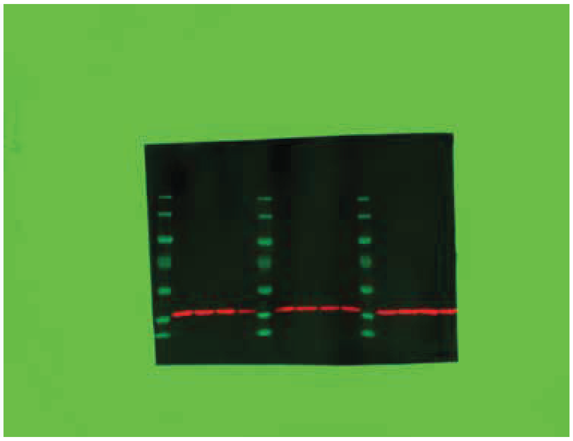

Cell lysates of HT-1080 WT and Gp78 KO cells transfected with siCTL or siRRBP1 were acquired in parallel with 3D STED experiments performed for Fig. 7 A,B and Western blotted in parallel with anti-RRBP1 (top) and anti-β-actin (bottom). Three biological replicates are shown and samples loaded as follows from left to right: HT-1080 WT siCTL; HT-1080 WT siRRBP1; HT-1080 Gp78 KO siCTL; HT-1080 Gp78 KO siRRBP1.

- Boxes show regions included in Supp Fig 3B.
- Fluorescent view of the blots including molecular weight markers (in green from top: 170, 130, 100, 70, 55, 40, 35 kDa) are shown to the right.
- RRBP migrates at ~180 kDa and β-actin at ~43 kDa.
